# Enhancement of the Mechanical Properties of Basalt Fiber-Wood-Plastic Composites via Maleic Anhydride Grafted High-Density Polyethylene (MAPE) Addition

**DOI:** 10.3390/ma6062483

**Published:** 2013-06-18

**Authors:** Jinxiang Chen, Yong Wang, Chenglong Gu, Jianxun Liu, Yufu Liu, Min Li, Yun Lu

**Affiliations:** 1 Key Laboratory of Concrete and Prestressed Concrete Structures of Ministry of Education & International Institute for Urban Systems Engineering, Southeast University, Nanjing 210096, China; E-Mails: kang2008year@126.com (C.G.); llljjjxxxun@126.com (J.L.); limin.li@163.com (M.L.); 2 Faculty of Mechanical Engineering & Automation, Zhejiang Sci-Tech University, Hangzhou 310018, China; E-Mail: dcuwy@163.com; 3 Jiangsu Key Laboratory of Construction Materials, School of Materials Science and Engineering, Southeast University, Nanjing 211189, China; E-Mail: yfliu@seu.edu.cn; 4 Department of Mechanical Engineering, Graduate School & Faculty of Engineering, Chiba University, Chiba 263-8522, Japan; E-Mail: luyun@faculty.chiba-u.jp

**Keywords:** WPC, basalt fiber, MAPE, composite material

## Abstract

This study investigated the mechanisms, using microscopy and strength testing approaches, by which the addition of maleic anhydride grafted high-density polyethylene (MAPE) enhances the mechanical properties of basalt fiber-wood-plastic composites (BF-WPCs). The maximum values of the specific tensile and flexural strengths areachieved at a MAPE content of 5%–8%. The elongation increases rapidly at first and then continues slowly. The nearly complete integration of the wood fiber with the high-density polyethylene upon MAPE addition to WPC is examined, and two models of interfacial behavior are proposed. We examined the physical significance of both interfacial models and their ability to accurately describe the effects of MAPE addition. The mechanism of formation of the Model I interface and the integrated matrix is outlined based on the chemical reactions that may occur between the various components as a result of hydrogen bond formation or based on the principle of compatibility, resulting from similar polarity. The Model I fracture occurred on the outer surface of the interfacial layer, visually demonstrating the compatibilization effect of MAPE addition.

## 1. Introduction

The study of natural materials has become increasingly important in recent years due to the growing awareness of the need to protect ecological and environmental resources, including shrinking forest resources [1]. Wood-plastic composites (WPCs) are made of plant fibers and thermoplastic materials and are fabricated by extrusion, injection, hot molding and other methods [2]. WPCs inherit the mechanical properties, color and texture of the wood type used [3,4], and they are therefore widely used in the construction industry and for home improvement, gardening, municipal, automobile and other applications such as pallet construction [5]. The raw materials used to manufacture WPCs can be obtained from waste plastic and scrap timber sources, with the result that the production and use of WPCs supports “turning waste into something useful” [6,7]. However, in comparison to actual wood, several disadvantages are associated with WPCs, including increased energy consumption during production, higher production costs and an approximate doubling in density with a simultaneous reduction in the specific strength per mass [8]. The uses of WPCs are restricted by their current mechanical properties; for example, wood-plastic materials have largely been confined to outdoor construction, which tends to be tolerant of the lesser mechanical properties of these materials. Until recently, WPCs could not be used as structural materials for applications that require better mechanical properties [9]. One possibility for improving the mechanical properties of WPCs is basalt fiber (BF). BF is colloquially known as the “21st-century nonpolluting green material”. BF is a new type of fiber prepared by drawing a natural ore, melted at a high temperature, through a platinum-rhodium alloy [10]. BF has numerous raw material sources, is inexpensive [11], and has excellent properties such as corrosion resistance, minimal moisture absorption and the ability to withstand high temperatures, provide thermal insulation, and absorb sound [12,13]. BF is also a cost-effective and high-strength material [14,15,16] that has been widely used in road construction [17], buildings and other applications that require reinforcement [18].

Many studies have attempted to overcome the shortcomings of WPCs [8,19], yielding gratifying results in terms of material formulation [20,21] and modification [22,23,24,25]. However, new breakthroughs are required to make WPCs more useful as structural materials. Woody materials contain a significant number of continuous fibers such as lignin fibers, and they have excellent mechanical properties, implying that engineers can learn a great deal from trees (natural wood). For example, wood-plastic composites could include high-strength fibers that are significantly longer than crushed wood grain fibers. Additionally, this type of composite can be expected to exhibit a good strengthening effect. In a previous investigation, BF with 3- or 12-mm-long fibers was directly added to a commercial WPC that had been previously characterized [26]. We found that the addition of BF led to a slight improvement in the tensile and flexural strength of the WPC but did not improve its elongation. Additionally, the mechanical properties of the BF-WPC exhibited poor stability. Wood fiber and BF, a type of inorganic fiber, are two distinctly different materials with unique physical and chemical properties [27,28]. The interface between these two materials can be significantly improved by the addition of a compatibilizer [16,29,30,31,32,33]. This study investigated the effects of maleic anhydride grafted high-density polyethylene (MAPE) on the mechanical properties of basalt fiber-wood-plastic composites (BF-WPCs) and studied the mechanisms of those effects by examining the interfacial fracture morphology and the physical and chemical interactions between the constituents. An approach is suggested for the development of high-strength, lightweight WPC structural materials by the application of bionic theory [34,35], and the results suggest a new strategy for the protection of precious ecological resources such as wood.

## 2. Experimental

### 2.1. Experimental Design and Materials

#### 2.1.1. Experimental Design

Based on the results of preliminary experiments [26], the BF was chosen to be 6 mm in length, and the BF content was selected to be 20 wt % of the total amount, including the BF and WPC fractions. The samples were divided into six groups based on the addition of MAPE, which ranged from a weight ratio of 0% to 12% in increments of 3% (denoted as 20%–0%, 20%–12% or abbreviated as 0%, 12%). The pure WPC sample with no BF or MAPE is denoted as 0%–0%.

#### 2.1.2. Experimental Materials

The WPC particles (teak powder/high-density polyethylene (HDPE) = 7:3) were produced by SHXINJIXIN Co., Ltd. (Shanghai, China). The BF (length: 6 mm, diameter: 17 µm) was produced by ZHEJIANG GBF BASALT FIBER Co., Ltd. (Jinhua, China), and the MAPE (type KT-12, with a 1.0%–11.5% grafting rate and a density of 945 kg/m^3^) was obtained from SHENYANG KETONG Plastic Co., Ltd. (Shenyang, China).

### 2.2. Sample Preparation, Material Property Testing and Equipment

#### 2.2.1. Sample Preparation

The BF and WPC were mixed for 10 min using a two-roll mill; the front roll was set to 160 °C and the back roll to 170 °C. The sample was then immediately hot-molded at 180 °C with a plate vulcanizer (type XLB-25 D, Shanghai First Rubber Machinery factory), preheated for 5 min and finally held at a pressure of 8 MPa for 10 min. Plates of the BF-WPC were then made for tensile and flexural testing. This approach differs from the previously published method [26] in which the sample was cooled for 12 h and then crushed.

#### 2.2.2. Mechanical Property Testing

The samples were prepared for tensile and flexural testing in accordance with GB/T 1040.2-2006 and GB/T 1449-2005, and the tensile and flexural tests were performed using an electronic universal testing instrument (REGER-200A, produced by Shenzhen REGER Instrument Co., Ltd. (Shenzhen, China)). The gauge length was chosen to be 80 mm for the tensile test and 60 mm for the flexural test. The test rates were all selected to be 10 mm/min. Five sample types were tested, and each sample type was analyzed three times. The total sample size was 15. The tests were executed three times, in April and December of 2011 and in February of 2012.

#### 2.2.3. Fractography

The fracture surfaces (fractography) were investigated with scanning electron microscopy (SEM: JSM-5610LV) after the sample sections had been vacuum-coated with gold.

## 3. Results and Discussion

### 3.1. Mechanical Properties of BF-WPC

**Figure 1:**
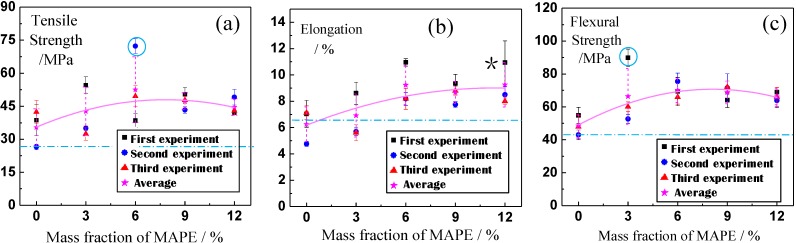
The relationship between the mechanical properties of the wood-plastic composite (WPC) and the mass fractions of basalt fiber (BF) and/or maleic anhydride grafted high-density polyethylene (MAPE) for (**a**,**b**) tensile properties and (**c**) flexural properties. Significant differences between the average values of different batches are marked as ○, and a large dispersion in the same batch (Figure 1b) is marked as *.

Figure 1 shows the average values measured in each of three independent experiments (marked separately with tetragonal, triangular, and circular symbols). The overall average values and the dispersion of the experimental results are shown in Figure 1 (marked with the star symbol). The solid line represents the regression curve of the overall average of the experimental results, while the overall average of the pure WPC sample without BF is shown as a dotted line for comparison. Comparison with the dotted line in Figure 1 demonstrates that MAPE addition improved the tensile strength and flexural strength of the BF-WPC, although it increased the elongation compared with the WPCs with the short BF and without MAPE. Several differences can be observed among these three independent experiments, with the solid lines in Figure 1 representing the average values of 15 datapoints (these results are discussed in further detail below). The mechanical properties of the BF-WPC with MAPE increased drastically with increasing MAPE content and reached their maximum values when the MAPE content was less than 6%. Based on the discrete nature of the experimental data, Student’s *t*-test was performed to determine whether the MAPE addition led to a significant change in the strength index. This test was performed by comparing samples with no MAPE (0% sample) to the sample with near maximal mechanical properties (6% sample). Table 1 presents the results of the *t*-test. The results are interpreted based on the criterion P (t_0_) ≤ 0.05, and significant results are labeled with a star (*). Compared with the sample with no MAPE (Table 1), the indexes of the other samples increased significantly, except for the experimental results of the sample with 3% MAPE content. Compared with the maximum value observed for 6% MAPE content, the tensile strength significantly decreases andthe remaining two indicators do not change with increases in MAPE content above 6%. Thus, the maximum enhancement of mechanical properties is achieved at a MAPE content of approximately 6%, with no obvious effect with a further increase in MAPE content. The maximum values of the tensile and flexural strengths were increased by more than one-third versus the WPC without the addition of MAPE and by more than one-half versus the pure commercial WPC. When the MAPE content was greater than 6%, the tensile and flexural strengths decreased somewhat as MAPE content increased, although the elongation improved slightly. These results are consistent with the previous report by Chen *et al.* [36] regarding the tensile and flexural strengths of the WPC, which reached maximum values at a MAPE content of 6%–8%.

**Table 1:** *t*-Test of the experimental results.

Index	Item	0%	3%	6%	9%	12%
Tensile strength	0%	–	0.28	0.00 *	0.00 *	0.00 *
	6%	0.00 *	0.01 *	–	0.17	0.01 *
Elongation	0%	–	0.69	0.00 *	0.00 *	0.00 *
	6%	0.00 *	0.00 *	–	0.44	0.97
Flexural strength	0%	–	0.00 *	0.00 *	0.00 *	0.00 *
	6%	0.00 *	0.42	–	0.96	0.09

Because the density of the BF (approximately 2600–3000 kg/m^3^) is significantly higher than that of the WPC (approximately 1 t/m^3^), the densities of the pure WPC samples were measured to determine whether the mechanical properties of the experimental WPC had improved. The density of samples without BF is 1.01, and BF and MAPE addition increases the density to 1.21, as determined by averaging measurements from five samples with 6% MAPE content. Therefore, the specific strengths were calculated for both the pure WPC and regression curve values at a MAPE content of 6%. The increasing rates of specific strength (IRSS) between the two groups can be calculated as follows: the tensile strength is of 43%, the elongation is of 7%, and the flexural strength is of 33%. The tensile and flexural strengths both increased by approximately one-third or more, while the elongation increased by less than 10%. Additionally, the specific strength value was improved.

### 3.2. Fracture Micrographs, a Model of the BF Interfacial Microstructure and its Mechanism of Formation

**Figure 2:**
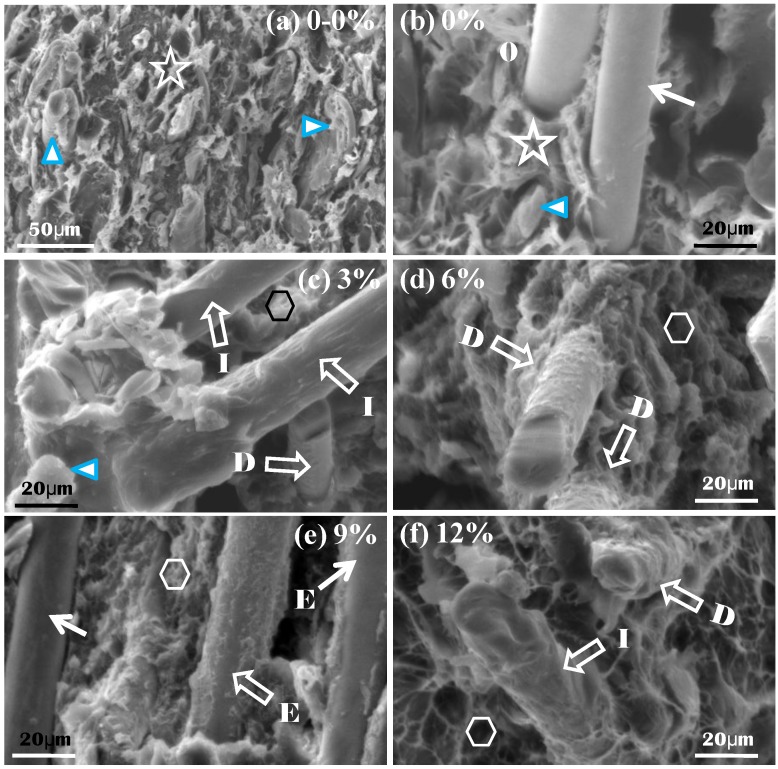
SEM micrographs of the tensile fracture surfaces of the composite: (**a**) pure WPC; (**b**–**f**) the WPC with the corresponding mass fractions of MAPE shown in each picture. The symbol “0” and the capital letters identify the interface mode; triangles: wood fibers, stars: HDPE; 0: the lack of an interfacial layer (IL), E: partial IL, D: wave-like IL, I: flat IL; arrows: smooth BF surface, wide arrow: ILs; hexagon: soft and dense surfaces.

Figure 2 presents the tensile fracture micrographs of each sample, revealing that the presence of MAPE changes the micrographs. The surface of the BF is smooth (Figure 2b), and the wood fiber and HDPE can be distinctly observed in the two samples that do not contain MAPE (Figure 2a,b: marked with a triangle and a star, respectively). The fracture surfaces exhibit tooth- or burr-like patterns. Overhanging basalt fibers (indicated with an arrow) protrude from the cross-sections, and their roots are entombed within the wood-plastic matrix, which contains BF in each sample (Figure 2b–f). The micrographs of the sample containing MAPE lead to the following observations: a small amount of the BF appears to be smooth (Figure 2e, marked with an arrow), but most of the BF has formed a boundary layer, and the shape of this layer is uneven (Figure 2d,e, marked with a wide arrow). Some of the BFs are relatively smooth, though they actually possess a thin membranous interfacial layer (Figure 2c,f, marked with a wide arrow). The wood fiber, HDPE and MAPE have nearly become integrated into a single material, and the cross-sections of the wood fiber and the HDPE have become soft and dense (Figure 2c–f, marked with a hexagon). Here, the burr-like patterns can no longer be observed.

Many previous studies have reported the modification of WPCs with a compatibilizer [37,38,39,40,41]. Although the type of compatibilizer (e.g., MAPP and MAPE) and wood fibers used in modified WPCs may differ, the strengthening mechanisms share some common characteristics. (1) A chemical reaction occurs between the active functional groups of the compatibilizer and the functional groups of the plant fiber [42], and this reaction forms either a chemical bond or a hydrogen bond, both of which commonly occur when polyethylene-based compatibilizer reacts with the hydroxyl groups [23] of the fiber or when the anhydride of MAPE [43,44,45] reacts with the glycosidic bond [46] (esterification)of the wood fiber; (2) This bonding reduces the polarity and hydrophilic nature of the plant fiber and improves the bonding strength between the wood fibers and the non-polar matrix, in accordance with the principle of compatibility between similar polarities; (3) The long chain within the compatibilizer (MAPE) can be inserted into the matrix, thereby enhancing the combination of the compatibilizer with the matrix as the molecular chain intertwines within the region of insertion [16,29,45]. Thus, the compatibilizer can effectively improve the interfacial bonding character of the composite by combining these three functions [30,47]. The findings of similar, previously reported experiments do not need to be repeated here, and the mechanisms by which the composite microstructure that has been integrated into one material and has become soft and dense upon the addition of MAPE are self-evident. Therefore, this paper continues to focus on the mechanism of enhancement by the MAPE compatibilizer.

**Figure 3:**
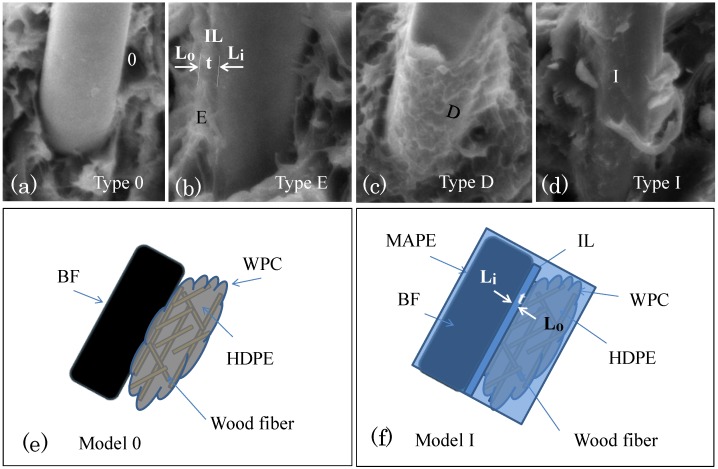
The interfacial layer between the BF and the WPC. (**a**–**d**) Interface types; (**e**,**f**) models before and after MAPE addition. IL: an interfacial layer, L_i_ and L_o_: the inner and outer faces of the IL, respectively; 0: the lack of an IL, E: partial IL, D: a wave-like IL, I: a flat IL.

Figure 3 presents fractographs showing several typical interfacial structures around BFs. Type 0 displays the smooth surface of the BF and the lack of an interfacial layer (Figure 3a); Type E represents the interfacial layer, IL, with its thickness marked as t, shown above a portion of the BF; and Types D and I are the same as Type E, except the interfacial layer is spread over the BF. The fracture behavior of Type D (Figure 3c) shows a wave-like mode that overlaps with the behavior of Type D; additionally, Type I shows a flat interfacial layer (Figure 3d). Table 2 summarizes the features of these types.

Based on the bonding mechanism and the effect of MAPE modification, the samples can be further divided into the two models model 0 and model I, as shown in Figure 3e,f. Model 0 corresponds to Type 0, which illustrates how the BF and WPC closely align even without an interfacial layer (Figure 3e). Type 0 can be found in all samples lacking MAPE and is sometimes found in samples containing MAPE. Model I corresponds to Types E, D and I. Model I is the primary interfacial model and can only be found in samples containing MAPE. The mark IL displayed in this model map represents the interfacial layer between the BF and the wood-plastic composite (Figure 3b–d,f). L_i_ and L_o_ represent the inner and outer faces of the interfacial layer, respectively. The plane, *i.e.*, the declining box, with a certain relative degree of declination and color-filling indicates the MAPE content and its modification function. The plane is the top layer and covers the BF and WPC contents, which indicates compatibilization by MAPE. The plane plays a blocking role, obscuring the boundary between the HDPE and the wood fiber. Both the BF boundary and the boundary between the HDPE and the wood fiber, which are presented in Figure 3f, are even more obscured than the boundary presented in Figure 3e.

**Table 2:** The features of the IL Types.

Type		0	E	I	D
Features	IL present?	No	Yes	Yes	Yes
	BF surface	Smooth	Partly covered	Fully covered	Fully covered
	IL shapes	(Nothing)	Lump or portion	Sheet, “I” shape	Wave-like, “D” shape

The approach used to construct Model I will now be further explained. As indicated earlier, the wood fibers have a substantial hydrophilic quality, and the HDPE resin is a hydrophobic polymer. The BF is an inorganic material formed by molten rock that also possesses inert properties. Therefore, without additives, the interface commonly associated with Model 0 (Figure 3a,e), *i.e.*, the interface between these three materials, is relatively weak due to the lack of chemical bonding among the active groups, which leads to poor compatibility. The MAPE added to the WPC [45] reduces the polarity and hydrophilicity of the wood fibers according to the three WPC strengthening functions mentioned above. According to the principle of compatibility based on similar polarity, MAPE not only enhances the compatibility and decreases the distance between the BF surface and the wood, but it also integrates itself into the BF surface, in addition to the nearly singular integration of the WPC. All of these factors improve the binding strength between the WPC and the BF [27,45,48]. Additionally, the Zeta potential determined by Hu *et al.* [49] demonstrates that the surface elements of the BF can form hydrogen bonds with the hydrophilic polar groups and that the BF surface contains a high Si content, which has the potential to chemically react with the surrounding active functional groups under certain conditions [28,32]. All of these factors contribute to the strengthening of the interfacial bond [16,29]. Furthermore, in addition to the increased compatibility between the BF and the WPC, closer proximity between the groups that participate in these hydrogen bonds or chemical reactions and the WPCs will facilitate hydrogen bond formation or chemical reactions [50,51]. This process of integrating all of the phases into one phase results in a large number of interfaces in Model I (Figure 2 and Figure 3). A more in-depth mechanism must be developed for future research.

### 3.3 The Relationships between the Mechanical Properties and MAPE Content

As previously mentioned, the BF-WPC composites were mainly composed of the four materials wood, plastic, BF and MAPE, which have significantly different physical and chemical properties. Initially, it can be difficult to quantitatively analyze such a complex composite material. Therefore, this section comprehensively explores the fracture morphology, the interfacial model and the relationship between these two factors, as well as the mechanical properties of the sample.

The fracture morphologies of the samples with MAPE indicate that the wood fiber, HDPE and MAPE fractions have become almost entirely integrated, forming a single material (Figure 2c,f). Additionally, the interfacial model developed for the BF surface shows that the vast majority of fibers exist as depicted in the Model I interface. If the wood plastic is the substrate (matrix) and the BF is used as a reinforcing fiber, then the matrix is integrated into a single material that should exhibit enhanced mechanical properties compared to that of the relatively loose matrix without MAPE. For the latter matrix, as shown in Figure 3b–d, all fractures of Model I occur at L_o_, and no fractures occur at L_i_. This result qualitatively demonstrates that the binding force of L_i_ is larger than that of the outer surface of L_o_. This result also demonstrates that MAPE addition improves the binding condition. Thus, a model is provided for the first time that shows the relationship between the MAPE, BF and WPC components, includes the concept of the interfacial layer, and includes sufficient schematic explanation to enable its modification [30,47]. This model provides evidence for the effect of compatibilization (Figure 1).

Future research questions include whether the MAPE additive has the same effect on the mechanical properties of the WPC samples and what MAPE content yields optimal results. A significant difference exists among the various constituents of the BF-WPC system with respect to its physical and chemical properties. Furthermore, the experimental results are also affected by the experimental conditions (e.g., the mixing uniformity and atmospheric conditions), which is why the experiment was repeated three times. These factors are more consistent within each experiment than among the three experiments, with little variation among experimental results from the same batch (with the exception shown in Figure 1b, marked as *), while the differences between the average values of the different batches are more significant (Figure 1a–c, marked as ○). Additionally, the differences between the overall averages for all three experiments are significant (Figure 1, dotted lines). Nevertheless, the average values obtained from the three separate experiments are taken to be more representative. As a further illustration of this point, it should be noted that the regression curve of the total average is relatively stable, as shown in Figure 1.

From Figure 2, in which the MAPE content is 6%, the matrix is not integrated into a single material, as larger lumps can also be identified (Figure 2d, identified as the hexagonal mark). However, at a MAPE content of 12%, a significant number of networked (wiry) shapes are visible, which appear to be the polymeric substance (Figure 2f, the hexagonal mark). This phenomenon is also reflected in the Model I interface (Figure 2d, the broad arrow marks). Overall, the thickness of the interfacial layer is thinner with a MAPE content of 3%, thicker with a MAPE content of 12%, and intermediate when the MAPE content is 6%. Because the tensile and flexural strengths of MAPE are largely similar to those of HDPE, the addition of large amounts of MAPE (9% or more) allows it to act as a matrix for HDPE, which decreases the quality of the interfacial layer [42,52], resulting in an excessively thick interface or a reduced strength. The tensile and flexural strengths also exhibit small decreases (solid regression line of Figure 1a–c). The MAPE itself exhibits good plasticity, and the elongation also exhibits a slight improvement (Figure 1b, solid regression line). The experimental results presented in Figure 1 show that the specific tensile and flexural strengths reach their maximums when the MAPE content is 5%–8%, although the elongation increases rapidly at first and then more slowly. These results are essentially the same as those obtained for the components integrated into a single material, possessing a BF with the “Model I” interface. A schematic diagram is presented first to illustrate the relationship between the MAPE with BF and WPC content as well as the concept of the interfacial layer and to intuitively demonstrate the effect of MAPE compatibilization.

Finally, it is worth noting the concern arising from the fact that the dispersion of each independent experiment is smaller than that of the total experiment and that the origins of this issue must be identified. This is a significant issue in terms of the possible future industrialization of BF-WPC composites. Although this issue was not fully solved in this work, we demonstrated that the MAPE additive was able to improve the mechanical properties of the BF-WPC. This issue of variability could be due to several preparation factors such as the composition of the BF-WPC complex, the preparation technology and the processing conditions. Thus, in the future, these issues can be addressed by designing comparative experiments. The results of this work therefore provide inspiration for future research efforts.

## 4. Conclusions

This investigation revealed that the MAPE content affects the mechanical properties of the BF-WPC, which was further discussed based on the fractographic analysis.

Explanations were proposed regarding the nearly complete integration of the wood fiber and the HDPE into each other when MAPE was added to the WPC and when the interfacial layer formed above the BF surface, as observed in the fracture micrographs. Two novel interfacial models were proposed and then used to explore the physical significance of MAPE addition. Intuitively, these explorations demonstrate the effect of compatibilization observed in the fracture micrographs, which further demonstrate that fracture occurs within the outer region of the interfacial layer when the sample contains MAPE. The interfacial mechanism of Model I was determined based on the formation of hydrogen bonds or chemical reactions among the components of the BF-WPC and on the principle of compatibility among similar polarities.

The fracture of the Model I interface occurred on the outer surface of the interfacial layer, visually demonstrating the compatibilization effect of MAPE. Investigating the matrix characteristics of the integrated material with different MAPE contents, in addition to the interfacial features of Model I, elucidated the qualitative relationship between the MAPE fraction and mechanical properties. The maximum specific tensile and flexural strengths can be achieved when the MAPE content is 5%–8%. The elongation increases rapidly at first and then slowly.
